# The highly polymorphic cyclophilin A-binding loop in HIV-1 capsid modulates viral resistance to MxB

**DOI:** 10.1186/s12977-014-0129-1

**Published:** 2015-01-09

**Authors:** Zhenlong Liu, Qinghua Pan, Zhibin Liang, Wentao Qiao, Shan Cen, Chen Liang

**Affiliations:** Lady Davis Institute, Jewish General Hospital, Montreal, Québec H3T 1E2 Canada; Key Laboratory of Molecular Microbiology and Biotechnology (Ministry of Education) and Key Laboratory of Microbial Functional Genomics (Tianjin), College of Life Sciences, Nankai University, Tianjin, 300071 China; Institute of Medicinal Biotechnology, Chinese Academy of Medical Science, Beijing, 100050 China; Department of Microbiology and Immunology, McGill University, Montreal, Québec H3A 2B4 Canada; Department of Medicine, McGill University, Montreal, Québec H3A 2B4 Canada; Current address: Département des Sciences Biologiques, Université du Québec à Montréal, Montréal, Québec H3C 3P8 Canada

**Keywords:** MxB, HIV, Transmitted founder virus, CypA

## Abstract

**Background:**

The human myxovirus-resistance protein B (MxB, also called Mx2) was recently reported to inhibit HIV-1 infection by impeding the nuclear import and integration of viral DNA. However, it is currently unknown whether there exist MxB-resistant HIV-1 strains in the infected individuals. Answer to this question should address whether MxB exerts an inhibitory pressure on HIV-1 *in vivo* and whether HIV-1 has evolved to evade MxB inhibition.

**Findings:**

We have examined ten transmitted founder (T/F) HIV-1 strains for their sensitivity to MxB inhibition by infecting CD4+ T cell lines SupT1 and PM1 that were stably transduced to express MxB. Two T/F stains, CH040.c and RHPA.c, were found resistant and this resistance phenotype was mapped to the amino acid positions 87 and 208 in viral capsid. The H87Q mutation is located in the cyclophilin A (CypA) binding loop and has a prevalence of 21% in HIV-1 sequences registered in HIV database. This finding prompted us to test other frequent amino acid variants in the CypA-binding region and the results revealed MxB-resistant mutations at amino acid positions 86, 87, 88 and 92 in capsid. All these mutations diminished the interaction of HIV-1 capsid with CypA.

**Conclusions:**

Our results demonstrate the existence of MxB-resistant T/F HIV-1 strains. The high prevalence of MxB-resistant mutations in the CypA-binding loop indicates the significant selective pressure of MxB on HIV-1 replication *in vivo* especially given that this viral resistance mechanism operates at expense of losing CypA.

**Electronic supplementary material:**

The online version of this article (doi:10.1186/s12977-014-0129-1) contains supplementary material, which is available to authorized users.

## Findings

Host restriction factors are important components of the antiviral defense. HIV research has led to the discovery of multiple restriction factors that not only inhibit HIV but also a wide range of other viruses. Examples of these restriction factors include APOBEC3G [1], Trim5α [2], tetherin [3,4], and SAMHD1 [5,6] (reviewed in [7]). A recent addition to this important family of proteins is human myxovirus-resistance protein B (MxB, also called Mx2). Despite the well-known antiviral property of MxA [8], no virus was known to be inhibited by MxB until 2011 when two screen studies showed that vesicular stomatitis virus (VSV), mouse herpesvirus type 68 (MHV-68) and HIV-1 could be restricted by MxB [9,10]. The anti-HIV-1 activity of MxB was further established by three independent studies which reported that MxB impedes HIV-1 DNA nuclear import and/or integration by targeting viral capsid [11-13]. A number of mutations in viral capsid were shown to render HIV-1 resistant to MxB, including N57S, G89V, P90A, N74D and others [12-15]. Furthermore, we identified in the escape viruses a capsid mutation A88T that overcomes MxB inhibition [11]. These results suggested that HIV-1 was able to escape MxB restriction by mutating its capsid sequence. However, the aforementioned capsid mutations are absent in the HIV sequence database likely because they all cause severe defects in virus infectivity. We speculate that, if MxB does exert a strong inhibitory pressure on HIV-1 infection *in vivo*, then there should exist MxB-resistant HIV-1 strains.

**Figure 1:**
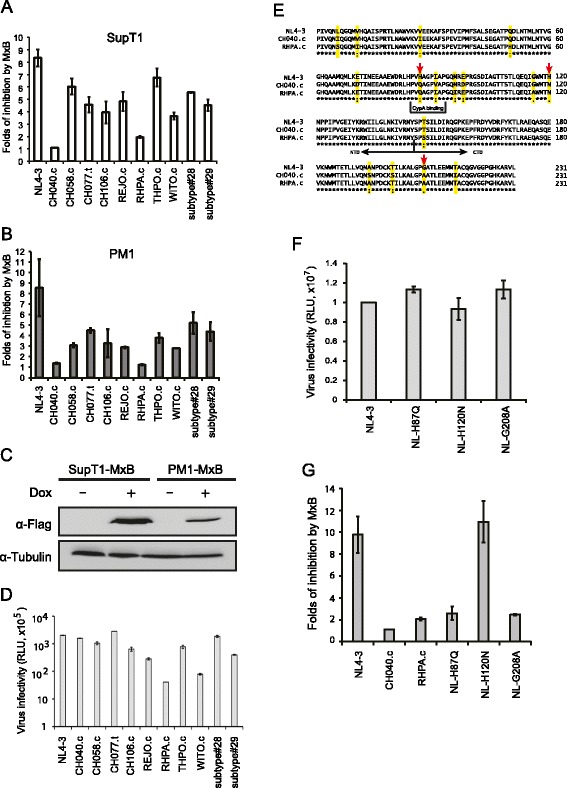
**Inhibition of transmitted/founder (T/F) HIV-1 strains by MxB. (A)** Ten T/F viruses were used to infect SupT1 cells that express MxB with doxycycline induction. The MxB protein has a C-terminal FLAG tag as described previously [11]. Levels of newly produced HIV-1 were determined by infecting the TZM-bl indicator cells. Folds of inhibition by MxB were calculated by dividing the amounts of HIV-1 produced from MxB-expressing SupT1 cells with virus amounts produced by control SupT1 cells. Results shown are the average of three independent experiments. The T/F viral DNA clones were obtained from the NIH AIDS Reagent Program [16,17]. **(B)** PM1 cells were stably transduced to express MxB under doxycyline induction, and were infected with the above ten T/F HIV-1 strains. MxB inhibition was determined as described above. **(C)** Levels of exogenous MxB-FLAG in the above SupT1 and PM1 cell lines, as determined by Western blotting. Tubulin was probed as the internal control. **(D)** Infectivity of the T/F HIV-1. The T/F viruses were generated by transfecting the HEK293T cells. Viruses of the same levels of reverse transcriptase (RT) activity were used to infect the TZM-bl indicator cells. The averages of three independent infections are shown. **(E)** Alignment of HIV-1_NL4–3_ capsid sequence with those of the two T/F viruses CH040.c and RHPA.c. Red arrows indicate the three amino acid positions that are occupied by the same amino acids in CH040.c and RHPA.c but by different amino acids in NL4-3. **(F)** Effects of mutations H87Q, H120N and G208A on the infectivity of HIV-1_NL4–3_. Virus infectivity was determined by infecting the TZM-bl cells. Averages of three independent infections are shown. **(G)** Mutations H87Q and G208A render HIV-1_NL4–3_ resistant to MxB. The mutants were tested in MxB-expressing SupT1 cells for their sensitivity to MxB inhibition.

To explore this possibility, we measured the effect of MxB on ten transmitted founder (T/F) HIV-1 strains. The T/F viruses were first produced by transfecting HEK293T cells with their proviral DNA constructs. Virus amounts were then determined by measuring viral reverse transcriptase (RT) activity. Viruses of equal values of RT activity were used to infect a SupT1 cell line that was stably transduced to express MxB under doxycycline induction [11]. Levels of viruses produced by these SupT1 cells were determined by infecting the TZM-bl indicator cells as previously described [11]. Consistent with what our group and others reported [11-13], MxB inhibited the HIV-1_NL4–3_ virus (a subtype B HIV-1 strain) by 8 to 10 fold (Figure 1A). The ten T/F viruses were inhibited to different degrees, with two strains CH040.c and RHPA.c exhibiting strong resistance to MxB (Figure 1A). We repeated this experiment in another CD4+ T cell line called PM1. Overall, the T/F viruses were less restricted by MxB in PM1 cells than in SupT1 cells (Figure 1B), which is likely attributed to the relatively lower expression levels of the exogenous MxB in PM1 cells (Figure 1C). Importantly, CH040.c and RHPA.c continued to resist MxB inhibition in PM1 cells (Figure 1B). The infectivity of the ten T/F viruses was also measured by infecting the TZM-bl indicator cells (Figure 1D), which showed a wide range of differences between the viruses. It is worth noting that the MxB-resistant strains CH040.c and RHPA.c differed in their infectivity by more than 30 fold. This suggests that the resistance of both viruses to MxB is independent of their infectivity levels.

Given the role of the viral capsid in countering MxB restriction [11-14], we aligned the capsid sequences of CH040.c and RHPA.c with that of NL4-3. Three amino acid positions in the capsid of both CH040.c and RHPA.c differ from those in NL4-3 capsid (Figure 1E). These are H87, H120 and G208 in NL4-3; Q87, N120 and A208 in CH040.c and RHPA.c. We next inserted Q87, N120 and A208 into NL4-3 capsid and generated mutations H87Q, H120N and G208A in order to determine which of these three amino acids lead to MxB resistance. None of these three mutations markedly affected the infectivity of NL4-3 (Figure 1F). When these NL4-3 mutants were tested in MxB-expressing SupT1 cells, H87Q and G208A, but not H120N, were refractory to MxB inhibition (Figure 1G).

**Figure 2:**
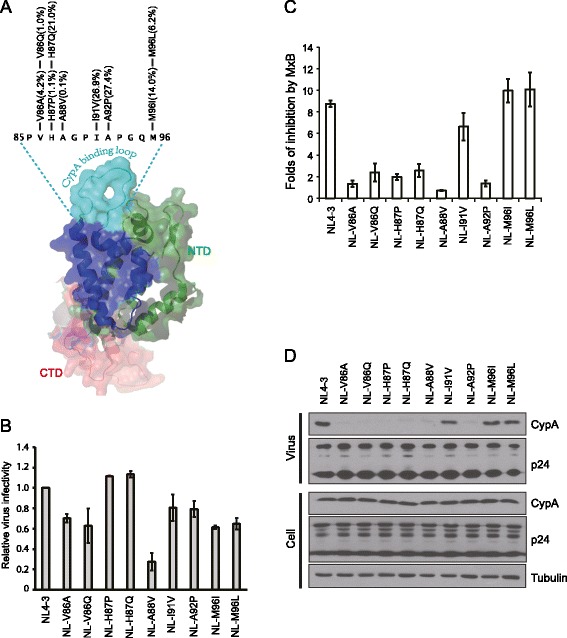
**Polymorphisms in the CypA-binding loop of the viral capsid modulate HIV-1 susceptibility to MxB inhibition. (A)** Illustration of the major polymorphisms in the CypA-binding loop of HIV-1 capsid. The prevalence of each variant was calculated on the basis of the capsid sequences (5372 entries) that are available at HIV database (http://www.hiv.lanl.gov/content/index). The position of CypA-binding loop is shown in the context of capsid structure (adapted from PDB ID 3P05) [19,20]. The capsid sequence from amino acid positions 85 to 96 is from the HIV-1_NL4–3_ strain. **(B)** Each of the nine major polymorphic amino acids was inserted into HIV-1_NL4–3_. The wild type NL4-3 and mutated viral DNA were transfected into HEK293T cells to produce progeny virus particles. Virus amounts were determined by measuring viral RT activity. Viruses of equal RT levels were used to infect the TZM-bl indicator cells. Results are summarized in the bar graph with the infectivity of wild type NL4-3 set at 1. **(C)** Wild type NL4-3 and its mutants were used to infect MxB-expressing SupT1 cells. Folds of inhibition by MxB were calculated as described in Figure 1A. **(D)** Incorporation of CypA into the wild type and mutated HIV-1_NL4–3_. Viral DNA was transfected into HEK293T cells. Virus particles in the culture supernatants were harvested by ultracentrifugation through a sucrose gradient as described in [21]. Virus amounts were determined by HIV-1 p24 ELISA. Virus particles of the same p24 quantities were examined in Western blotting for the presence of CypA. Lysates of transfected HEK293T cells were also subject to Western blotting to assess levels of endogenous CypA and viral Gag/p24 expression.

H87 is located in the cyclophilin A (CypA)-binding loop of HIV-1 capsid (Figure 2A) [18]. Q87 represents a major variant at this position of capsid in the circulating HIV-1 strains, with 21% representation in the HIV database (Figure 2A) (http://www.hiv.lanl.gov/content/index). This prompted us to test the resistance of other major amino acid variants in the CypA-binding loop, including V86A (4.2%), V86Q (1.0%), H87P (1.1%), A88V (0.1%), I91V (26.9%), A92P (27.4%), M96I (14%) and M96L (6.2%) (Figure 2A). We inserted each of these eight capsid variants into NL4-3. Except for A88V that reduced virus infectivity by 4-fold, the other mutations were well tolerated by HIV-1_NL-3_ (Figure 2B). We then used these viruses to infect MxB-expressing SupT1 cells and observed that although the I91V, M96I and M96L mutants were as sensitive to MxB inhibition as the wild type NL4-3 (Figure 2C), the mutants V86A, V86Q, H87P, H87Q, A88V and A92P were all resistant to MxB (Figure 2C). Therefore, at least six naturally existing amino acid variants at four positions in the CypA-binding loop render resistance to MxB.

We have previously shown that either knockdown of CypA or cyclosporine A (CSA) treatment to block CypA rescued HIV-1 infection of MxB-expressing SupT1 cells [11]. We therefore asked whether the above capsid variants in the CypA-binding loop modulated HIV-1 susceptibility to MxB by altering the binding of CypA to the viral capsid. To answer this question, we transfected the wild type and mutated HIV-1_NL4–3_ DNA into HEK293T cells and purified HIV-1 particles by ultracentrifugation through a sucrose gradient [21]. The presence of CypA in HIV-1 particles was examined by Western blotting and the results showed that the wild type NL4-3 and MxB-sensitive mutants I91V, M96I and M96L packaged substantial amounts of CypA as opposed to the MxB-resistant mutants V86A, V86Q, H87P, H87Q, A88V and A92P that packaged little or no CypA (Figure 2D). This data suggests that some HIV-1 strains have evolved to escape MxB inhibition at the expense of losing viral capsid binding to CypA.

**Figure 3:**
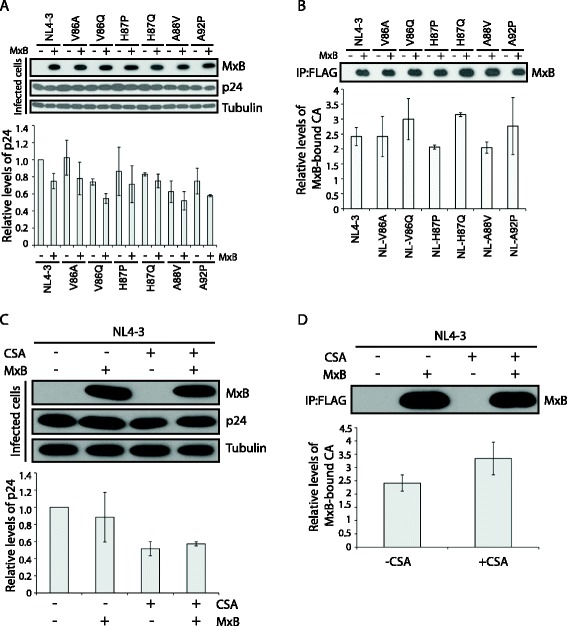
**Association of wild type and mutated HIV-1**_**NL4–3**_ **capsid with MxB.** Wild type NL4-3 or its mutants V86A, V86Q, H87P, H87Q, A88V and A92P were used to infect MxB-expressing SupT1 cells or control SupT1 cells without MxB expression. At 16 hours after infection, cells were harvested, suspended in a hypotonic buffer containing 10 mM Tris–HCl (pH8.0), 10 mM KCl and 1 mM EDTA, and lysed with 15 strokes in a 7 ml Dounce homogenizer. After clearing cell debris by centrifugation at 3,000 rpm for 5 min at 4°C, cell lysates were incubated with the anti-FLAG M2 agarose (Sigma) for 4 hours at 4°C. After extensive washing, the bound MxB was eluted with 3xFLAG peptide (Sigma) and the amounts of associated HIV-1 p24 were determined by ELISA. **(A)** Levels of MxB-FLAG, wild type and the mutated HIV-1 p24/capsid in the infected SupT1 cells as determined by Western blotting. Amounts of wild type and mutated p24 were also quantified by ELISA and results are shown in the bar graph. **(B)** Levels of wild type and mutated p24 that were associated with MxB. Levels of the eluted MxB-FLAG were assessed by Western blotting. For each virus, the amounts of viral p24 eluted from the M2 agarose were determined by ELISA, then calibrated by p24 amounts in the corresponding cell lysates. Levels of MxB-associated p24 were determined by dividing the values of M2 agarose-associated p24 from MxB-expressing cells with those of M2 agarose-associated p24 from the control cells. Results shown are the average of three independent infection experiments. **(C)** Levels of viral p24 in NL4-3 infected SupT1 cells under treatment of CSA (5 μM). **(D)** Effect of CSA treatment on p24 association with MxB. Detailed legend refers to **(B)**.

Recent studies reported the association of MxB with *in vitro* assembled HIV-1 capsid [22-24]. Therefore, in addition to modulating the interaction of CypA with HIV-1 capsid, the V86A, V86Q, H87P, H87Q, A88V and A92P mutations may also affect the binding of MxB to the viral capsid. To test this possibility, we infected MxB-expressing SupT1 cells or the control SupT1 cells with wild type HIV-1, or the above six MxB-resistant mutants. The MxB protein was then immunoprecipitated, and the amount of MxB-associated HIV-1 p24/capsid was determined by ELISA. The results revealed that MxB was associated with HIV-1 capsid in the infected SupT1 cells, albeit that both the wild type and mutated viral capsids were associated with MxB to similar degrees (Figure 3A and B). In addition, treatment with CSA did not diminish this interaction (Figure 3C and D). This observation corroborates prior results showing that neither mutations G89V and P90A in the CypA-binding loop nor CSA treatment altered the association of MxB with the *in vitro* assembled HIV-1 capsid complex [23,24].

In conclusion, we have identified two T/F HIV-1 strains that are resistant to MxB restriction in SupT1 and PM1 cells, and further mapped the resistant mutations to viral capsid. Our data also revealed the high prevalence of MxB-resistant mutations in the CypA-binding loop of the viral capsid. Since these MxB-resistant mutations diminish the association of viral capsid with CypA which acts as a host dependency factor for HIV-1, there is a cost for the virus to adopt this MxB-resistant pathway, which helps to explain why these MxB-resistant mutations have not prevailed the circulating HIV-1 strains. Together with the recent finding that the P207S, G208R and T210K mutations confer resistance to MxB [14], our data on the G208A mutation reinforces the importance of this region of capsid in modulating HIV-1 sensitivity to MxB. Considering that the A208 residue of capsid has a 35% prevalence rate in the circulating HIV-1 strains (http://www.hiv.lanl.gov/content/index), the G208A variant may serve a distinct mechanism, in addition to the variants in the CypA-binding loop, that HIV-1 employs to escape from MxB restriction *in vivo*.
